# The effect of family-based multidisciplinary cognitive behavioral treatment in children with obesity: study protocol for a randomized controlled trial

**DOI:** 10.1186/1745-6215-12-110

**Published:** 2011-05-06

**Authors:** Rimke C Vos, Jan M Wit, Hanno Pijl, Carolien C Kruyff, Euphemia CAM Houdijk

**Affiliations:** 1 Department of Pediatrics, Juliana Children's Hospital/ HagaHospital, the Hague, The Netherlands; 2 Department of Pediatrics, Leiden University Medical Center, Leiden, The Netherlands; 3 Department of Endocrinology and Metabolism, Leiden University Medical Center, Leiden, The Netherlands; 4 Psycho-medical Health Centre de Jutters, the Hague, The Netherlands

## Abstract

**Background:**

The prevalence of childhood obesity has increased rapidly during the last three decades in the Netherlands. It is assumed that mainly environmental factors have contributed to this trend. Parental overweight and low social economic status are risk factors for childhood obesity. Childhood obesity affects self-esteem and has negative consequences on cognitive and social development. Obese children tend to become obese adults, which increases the risk for developing cardiovascular complications, type 2 diabetes mellitus, and psychosocial problems. Additionally, the secretion of several gastrointestinal hormones, responsible for appetite and food intake, is impaired in obese subjects. Weight reduction through lifestyle changes in order to change health risks is, until now, suggested as the preferred treatment for childhood obesity.

The objective of this study is the effect evaluation of a family-based cognitive behavioral multidisciplinary lifestyle treatment. The intervention aims to establish long-term weight reduction and stabilization, reduction of obesity-related health consequences and improvement of self-image by change of lifestyle and learning cognitive behavioral techniques.

**Study design/Methods:**

In this randomized clinical trial newly presented children with obesity (8-17 years old) are divided, by randomization, in an intervention and control group, both consisting of 40 obese children. The intervention is carried out in groups of 8-11 children, and consists of respectively 7 and 5 separate group meetings for the children and their parents and 1 joint group meeting of 2 ½ hours. Main topics are education on nutrition, self-control techniques, social skills, physical activity and improvement of self-esteem. The control group is given advice on physical activity and nutrition. For normal data comparison, data were collected of 40 normal-weight children, 8-17 years old.

**Discussion:**

Because of the increasing prevalence of childhood obesity and the impact on the individual as well as on society, prevention and treatment of obesity in children is of great importance. For evaluation of short- and long-term effects of the treatment, measurements are taken before and after 3 months of treatment, and after 12 and 24 months follow-up. During these visits clinical and biochemical data are determined, cardiovascular fitness tests are performed and quality of life questionnaires are completed.

**Trial registration:**

International Standard Randomised Controlled Trial Number Register ISRCTN36146436

## Background

During the last three decades the prevalence of childhood obesity has increased dramatically in western countries, including the Netherlands [1-7]. In the Netherlands the highest prevalence of obesity is found in children and adolescents of Turkish and Moroccan origin [8,9]. Other risk factors for developing obesity include parental overweight or obesity, low socio-economic status, and low parental education level [4,9-11].

The adult cut-off values of BMI for overweight and obesity are 25 and 30 kg/m^2^, respectively, and are related to increased risk for morbidity and mortality [1]. However, in children BMI is age and gender dependent, so that BMI is usually expressed as standard deviation score (SDS). The working group on childhood obesity of the International Obesity Task Force (IOTF) determined cut-off lines for overweight and obesity in children based on the BMI-SDS that ended at 25 and 30 kg/m^2^, respectively, at 18 years of age, obtained from 6 large growth studies carried out before the obesity epidemic [2].

The rapidly increasing prevalence of childhood obesity seen during the last few decades, is mostly the result of an increased food consumption and a change from a more physically active lifestyle to a more sedentary one. Studies indicate that 50% of the children and 80% of the adolescents with obesity will remain obese in their adult life [12-14]. In adults obesity is associated with a higher risk for developing type 2 diabetes mellitus and cardiovascular disease [15-19] and in children obesity is associated with increased prevalence of hypertension, dyslipidemia and impaired glucose metabolism [20-27]. The clustering of these risk factors is called the Metabolic Syndrome (MS) also known as Syndrome X or the Insulin Resistance Syndrome [28].

For adults various definitions of the MS have been described. The most commonly used definitions are those proposed by the World Health Organization [29], the National Cholesterol Education Program's Adult Treatment Panel III [30] and the International Diabetes Federation [31]. These three definitions concur for the essential components (central obesity, hypertension, impaired HDL-cholesterol (HDL), triglycerides (TG) and impaired glucose tolerance). For the pediatric age group several definitions have been proposed, but modifications of the three mentioned definitions for adults are used most often [32-38]. However, consensus on the cut-off levels of the components of the MS for pediatric patients is even more difficult to obtain than for adult patients. One of the reasons may be that in children these cut-off levels are not only influenced by gender and ethnicity, but also by age and pubertal stage. In order to get better insight in the impact of the MS in the pediatric age group an internationally accepted definition is necessary.

Moreover, it has become clear that the adipose tissue is an endocrine organ [39,40]. It does not just serve as a fat deposit but it secretes a wide range of hormones and other proteins. Obesity represents a chronic state of inflammation, with increased levels of the C-reactive protein (CRP) [41-43] and decreased levels of adiponectin [44-47]. This chronic state of inflammation may be the common soil for the development of insulin resistance and cardiovascular disease. It has been shown that obese subjects, both adults and children, have significantly lower levels of adiponectin compared to normal weight individuals [44-49]. After clinically relevant weight loss, levels of adiponectin increase accompanied by improvement of insulin sensitivity [48,50].

The insulin secretion after consumption of nutrients is regulated by neural and hormonal signals from the gut and the intestine [51,52]. The gastrointestinal tract is the largest endocrine organ responsible for the regulation and signalling response of food intake to the brain [51,52]. Gastrointestinal hormones are the key mediators in these processes of food intake regulation. Several gastrointestinal hormones are impaired in obese persons, including ghrelin [51-56], peptide YY (PYY) [51,52,57-59] and glucagon-like peptide 1 (GLP-1) [52,60-64]. In contrast to many other endocrine hormones, gut hormones have predominantly short-term actions [52].

Ghrelin is a 28 amino acid peptide released from X/A-like cells of the gastric oxyntic glands, and in lower amounts from the small intestine and the hypothalamus [51-53]. Ghrelin is the only gut hormone known to increase food intake. An inverse association is found between circulating levels of ghrelin and BMI, so that obese subjects have lower levels of ghrelin [51-54,65-67]. There is still much uncertainty about the effect of weight loss on the ghrelin response. Some studies found increased ghrelin levels after weight lost [51,52,56], but others could not confirm this finding [56,68].

Both PYY and GLP-1 are synthesised and released after food intake by L-cells of the distal gut [51,52,57,59,69]. For fasting PYY an inverse association is found with BMI, in both adults [51,52] and children [51,52,57,59], whereas no significant association with body weight was found for fasting GLP-1 [52]. Postprandial levels of both hormones [51,52,59,52-64,70] are found to be blunted in obese individuals, and weight loss seems to affect this [59,52,61,71]. However, the mechanisms underlying the synthesis and release of especially GLP-1 from L-cells are largely unknown.

Besides these physiological changes, obesity also has a huge impact on the psychosocial well-being of the child with obesity [7,72-75]. Obesity is often associated with feeling ugly, lazy, stupid and with low self-control, which results in a negative self-image and, consequently, social isolation of the obese child. This social isolation can aggravate the obesity because the child can use eating as a coping strategy [72,76].

The question which intervention is most effective in treating both the physiological and psychological aspects of childhood obesity has not yet been definitely answered. The recently published update of the Cochrane review 'Interventions for treating obesity in children' [77] included 54 randomized clinical trials on lifestyle interventions, with 36 focusing on (family-based) behaviorally orientated treatment programs. From the 54 included studies on lifestyle interventions, 40 interventions lasted longer than 6 months, 6 of which up to one year and 4 lasting 2 years. The aim of this Cochrane review was to ascertain which intervention is most effective in the treatment of childhood obesity. However, study design, duration of follow up and outcome measures differed significantly in the included studies and it was only possible to give a recommendation. The results of the meta-analysis showed that for the treatment of childhood obesity a behavioral lifestyle intervention, combined with parental involvement, is preferred over standard care or self-help.

The intensity of the intervention appears important for the efficacy, because an observational study, including 129 treatment centers in Germany, Austria and Switzerland [78] demonstrated a significantly positive association between BMI-SDS reduction and intensity of the intervention after 24 months follow-up, while there was no association with duration. These authors also found a reduced BMI-SDS in nearly half of the children after 24 months of follow-up, with 25% of them showing a reduction of > 0.5 SDS, which has been demonstrated to be clinically relevant [79-81]. Younger children (< 12 years) showed significantly higher success rates at 24 months' follow-up.

A major problem with lifestyle intervention programs is the high percentage of incomplete follow-up data due to drop-out or lack of documentation, which makes it very difficult to reliably assess the effectiveness of the treatment. Therefore, in order to accomplish long-term weight loss and health improvement, future research should focus on adequately powered long-term (> 6 months) evaluation of lifestyle interventions, with psychosocial determinants for behavioral change and parental involvement.

### Objective

The primary aim of this study is the effect evaluation of a family-based multidisciplinary cognitive behavioral treatment on obesity (expressed as body mass index (BMI)-standard deviation score (SDS)) compared to standard care (advice on increased physical activity and dietary changes) in children with obesity. The secondary aim of the study is to investigate the effect of this treatment on changes in waist circumference, insulin sensitivity, inflammation, secretion of gastrointestinal hormones and changes in physical fitness and quality of life, compared to standard care. Furthermore, we aim at assessing the effects of possible predictive factors for the long-term response to treatment.

### Main research question

1. Do children (8-17 yr.) with obesity show a significant decrease (> 0.5) in BMI-SDS (as defined by Cole et al. [82]) after 3 months of intensive treatment compared to children who were given advice on increased physical activity and dietary changes?

### Secondary research questions

1. Do children with obesity have significantly more beneficial effect of 3 months intensive treatment compared to standard care on changes in waist circumference, insulin sensitivity, secretion of gastrointestinal hormones, cardiovascular fitness and quality of life?

2. Will the hypothesized beneficial effects of 3 months intensive treatment in children with obesity persist after 12 and 24 months of follow-up?

3. Are ethnic background of the children, the level of parental education, or the socio-economic status predictive variables for the degree of obesity in these children, and for the success of treatment?

## Methods/Design

### Design

The study design is a randomized clinical trial for 2 years.

Newly presented children with obesity referred to the pediatrician are physically examined. If a child meets the inclusion criteria of the study, the pediatrician informs the parents and the child about the study. After written informed consent, the children are randomized to the intervention and control group, after stratification for gender and ethnicity, in two age groups, one from 8 to 12 years and one from 13 to 17 years. An experienced assistant blinded for the study design measures weight and height.

Ethnicity is determined according to self-reports by the parents. For this analysis children are classified as 'Northern European' if both parents report the Northern European ethnicity, and 'Others' if one or both parents report another ethnicity.

To enable comparison of the participants' anthropometric and biochemical data with proper reference data, an age-matched sample of healthy children with normal weight is recruited from the youth health services (Jeugd GGD Haaglanden).

Measurements are taken before (t = 0 months) and after (t = 3 months) the treatment of the intervention group and after a follow-up period of 12 months. For long-term purposes the intervention group is also evaluated after 24 months. The control group is offered the same treatment regimen after 12 months.

The normal weight control group is measured once.

### Eligibility criteria

Children with obesity (according to Cole et al. [2]) aged 8-17 years, living in the Hague and in the area around the Hague and referred to a pediatrician, are invited to participate. Reasons for referral are overweight or obesity, and increased risk of co-morbidity (e.g. hypertension, family history of diabetes mellitus and/or hypercholesterolemia and/or cardiovascular disease before the age of 55, Hindustani ethnicity).

Potential participants are excluded if their knowledge of the Dutch language, intelligence or social skills are insufficient to participate in the group. Other exclusion criteria are use of medication that might have an effect on weight loss, medical co-morbidities that could affect participation, or previous enrollment in another cognitive behavioral treatment program with the focus on reducing obesity.

### Recruitment

During the first pediatric consultation inclusion and exclusion criteria are checked. Motivation and expectations of both children and parents are evaluated; e.g. it is checked whether they understand the reason to participate and know why it is important to reduce body weight, and whether their expectations of the treatment are realistic. Pros and cons of treatment versus no treatment are discussed, as well as alternative treatment options. If the children do not fulfill the inclusion or exclusion criteria an alternative treatment is offered to the parents and the child.

### Randomization

For the children who meet the inclusion criteria the parents and the children are asked to sign the informed consent. Following informed consent of all participating children and parents the children are stratified by gender and ethnicity ('North European' and 'Other') and randomized to the intervention or control group according to coin-tossing. In order to obtain a similar size of the intervention and control groups, blocked randomization is applied with an allocation ratio of 1:1. Randomization is carried out by a member of the team who does not take part in the treatment. Both the intervention and control groups consist of 40 children per group. The children in the intervention group are divided into smaller groups of 10, depending on the child's age. After randomization, children receive a randomization code. All data are analyzed according to this randomization code. The key to the source data is only known by the researcher and research coordinator. The researcher is familiar with the study procedure, is trained prior to the study and collects all data for this study. The normal-weight control group consists of 40 children, 8-17 years, with normal body weight.

### Ethic Aspects

This study is conducted in agreement with the 'Declaration of Helsinki'.

Approval is obtained from the regional medical ethical committee Zuid-West Holland. All parents and children gave their written informed consent.

### Treatment

#### Treatment Intervention group

The cognitive behavioral treatment program consists of an intensive phase of three months, followed by booster sessions for a total period of two years. During the intensive phase of the program the intervention group is offered 7 group meetings of 2½ hour and the parents are offered 5 separate parent meetings and 1 meeting together with the children.

The primary goal of the cognitive behavioral treatment is recognition and acknowledgement of the obesity and learning a healthy lifestyle. This is accomplished by health education and by teaching cognitive behavioral techniques.

The secondary goals of the treatment program are a reduction of 10% of the body weight during the intensive phase of the treatment and a further reduction or at least maintenance of the reduced body weight.

A treatment period of at least two years is chosen because it will take time to adapt to a new lifestyle and the risk of relapse is considerable.

##### Screening phase

During the screening phase the children with their parents are seen at two separate occasions individually by a dietitian, a child-physiotherapist, a child-psychologist and a social worker. In this way it is evaluated whether the timing of the treatment is appropriate, and whether there are individual family situations which could interfere with the treatment. Also causal factors of the obesity are discussed and opportunities to establish lifestyle changes are mapped out.

##### Dietitian

During the first consultation the weight and diet history of the child and their family are verified. Also their general knowledge of nutrition and their expectations of the role of a dietitian in helping them to reduce body weight are discussed. At the end of the session the child is asked to make a diet report of two weekdays and one weekend day. During the second session an evaluation of the diet reports is made and information is provided about nutrition and healthy eating behavior according to the traffic light nutritional list. The traffic light nutritional list [83] is divided into several main food groups (e.g. fruits, vegetables, grains, milk and other dairy products, meat, fish, and others). Foods within each group are color coded according to the calorie density per average serving and Dutch standards for health nutrition. The colors, similarly to those of a traffic light, are green for "go", orange for "approach with caution", and red for "stop". The goal of the use of the traffic light nutritional list is not only to reduce calorie intake but more importantly to learn healthy eating behavior. At the end of the second session achievable treatment goals are formulated.

##### Physiotherapist

Current physical activity level and sedentary behavior of the child is reviewed. Energy intake versus energy expenditure is evaluated and visualized for the child by a computer program. With this computer program the child can choose a favorite product from a nutritional list and the program then shows the child the amount of time and intensity of physical activity that is necessary to burn the energy consumed. Also options to change or optimize physical and sedentary activities are debated. The child is advised how to find suitable exercise programs.

##### Psychologist

Most children with obesity do not have sufficient insight in how obesity develops and how to reduce body weight. The role of the child psychologist is to help the child not only to reduce weight by learning cognitive behavioral techniques, but also to deal with and accept their own body.

For the success of the treatment the children must be motivated and able to change behavior. It is explained that the role of the parents during the treatment is that of 'the therapeutic helper'. The degree of suffering with regards to body image and the role of eating and obesity in the family are evaluated as well.

During the individual consultations by the child psychologist several questionnaires are asked to be filled; the 'Dutch Eating Behavior Questionnaire' (Nederlandse Vragenlijst Eetgedrag/ NVE) [84], the 'Child Behavioral Checklist' (CBCL) [85], the 'Youth Self Report' (YSR) [85], the 'Teacher Report Form' (TRF) [85] and the 'Perceived Competence Scale for Children' (CompetentieBelevenisSchaal voor Kinderen/ CBSK) [86].

Before the child starts with the group sessions individual treatment goals are written down in a contract. It is important that the goals are achievable to avoid disappointment. Also a buddy is chosen by the child who will help to achieve his or her goals.

##### Treatment

Group treatment is a useful way to learn cognitive behavioral techniques especially if groups are limited to 8-10 participants. Children can learn from each other (modeling). Peer group support is especially important for the older children group (≥ 12 yrs).

Before the first group session the children receive a treatment manual with the objectives and goals of the treatment and, per session, information about the topics discussed. To achieve successful treatment results it is important, at the beginning of the treatment, to focus more on the effort to change habits and the input of the participants and less on the weight reduction goals. This is accomplished by making the child aware of its own actions and way of living that has led to his or her obesity.

Several cognitive behavioral techniques are learned (knowledge, skills and attitude) during the intensive phase of the treatment in order to maintain long-term lifestyle change and body weight reduction. The cognitive behavioral strategies are learned during six sessions of 2½ hours per session. Sessions are given biweekly.

Each session contains the following components:

• Determination of body weight

• Homework discussion and evaluation

• Education

• Physical activity

• Role playing

• Discussing homework for next meeting

• Set goals linked to educational topics of the session

##### Parental Involvement

Not only the motivation to change the lifestyle of the children is important, but also the motivation of the parents. Parents must be willing to invest time in the treatment. Separate parallel parent group sessions (5 evenings) are offered by a dietitian and a social worker. In most cases parents do the shopping and food preparation, so for the treatment to be successful parental involvement and support is essential.

The role of parents is that of 'the therapeutic helper' who gives positive feedback to their children and supports the child in a positive way.

Parents should be a role model for their children, giving a good example through eating healthy food, increasing physical activity and decreasing sedentary activity.

##### Children's Group Sessions

##### Educational topics in session 1

Most children with obesity have negative experiences with group activities. For example, they are often not included in social events or chosen last by peers during sport activities. Therefore, during the first session much time is spent in getting acquainted with each other. A good group bond is important for the effect of the treatment because peer support can be very helpful in the treatment of obese children. During this part of the session children give their individual motivation for participating.

The topic of the second part of this session is nutritional information and the balance between energy intake and energy expenditure. This is explained by using the symbol of a balance. Solutions to improve the misbalance are discussed. When the energy intake part of the balance is greater than the energy expenditure part, energy expenditure must be increased or energy intake must be decreased, or both.

##### Educational topics in session 2

The main topic of the second session of the treatment phase is again information on healthy nutrition. Different categories of nutrition and quantities of each category linked to a healthy eating pattern are discussed. Children learn how to read product labels and to obtain information on misleading advertisement. They also discuss how to deal with meals (breakfast, lunch, diner, 2 healthy snacks), and how to make healthy choices and develop healthy eating habits (small bits, slow eating, eating at the family table, no other activities during eating). In games the educational topics are rehearsed and participants need to label products according to the traffic light method [83].

##### Educational topics in session 3

In this session self-control techniques to cope with difficult situations are taught. Children formulate and acknowledge difficult situations (e.g. birthday parties, holidays, lunch breaks at school, being at home alone). Problem solving alternatives are debated (e.g. to avoid a situation, doing something else, participate in a situation and eat less, or participate followed by extra exercise afterwards). The problem-solving alternatives are rehearsed with role-playing.

Other psycho-educational topics reviewed during the third session are self-reward (when coping well with a difficult situation) and self-regulation situations (making a plan how to integrate healthy behavior in daily living). Stimulus control is also one of the psycho-educational topics of the third session (remove unhealthy stimuli at home, encourage healthy behavior, eat at the dinner table, reduction of environmental stimuli linked to eating)

##### Educational topics in session 4

Most people associate obesity with being ugly, lazy or stupid which makes children with obesity often the subject of teasing by peers. The topics of the fourth session are therefore coping with teasing and being teased, how to deal with it and how to react with dignity. The children are explained that there are three coping strategies to deal with this situation; react angry (fight), walk away (flight) or deal with the situation (preferred strategy). Using role-playing the children are taught to react with a dignified, but powerful response. During this session the children are asked to think of someone in their environment who can help them during difficult situations or when they are teased.

##### Educational topics in session 5

The focus of the fifth session is self-image. Most children with obesity have a low self-image and a low health-related quality of life. Not only other people associate obesity with being ugly, lazy or stupid, but the children also think likewise about themselves. It is difficult for them to mention positive things about themselves. Children are taught to take a positive look at themselves and name positive, nice things or characteristics about themselves and other children in the group.

##### Educational topics in session 6

During the sixth session the children are given the opportunity to repeat topics discussed during the other sessions. Also cognitive strategies and relapse techniques are taught to maintain newly learned behavior.

##### Parent Group Sessions

##### Session 1

Parents are given information on the treatment program of the children and what they can expect of their own group sessions. Information on healthy nutrition, product information, quantities, eating moments, eating locations, how to help their children (also when there are children in the family with a normal body weight) are discussed. Parents also receive advice on parenting styles to give strict rules but also manage the family in a pleasant way.

##### Session 2

During the second session parents are taught how to support their children during the treatment and thereafter by acknowledging the child's efforts and giving positive feedback. It is made clear to the parents that for the treatment to be a success, parental support is very important.

##### Session 3

Parenting styles are taught during the third session on how to set boundaries and how to limit the sometimes abnormal eating behavior of their child.

##### Session 4

During this session topics of the previous sessions are repeated, and questions relevant to the obesity subject are answered.

##### Session 5

During the last separate parental group session a therapist discusses the role of all other family members with regard to the treatment in the family (e.g. are other family members supportive, how do they cope with the lifestyle changes?).

##### Joint Session

During the last session children and parents celebrate the end of the intensive period in a healthy way. Children are asked to make healthy snacks and in this way the children can bring into practice how to cope with a normally difficult situation. Games and activities are planned as well, so children and parents can bring into practice that festive parties can also be associated with fun and social interaction instead of the intake of food alone.

##### Follow-up

Booster sessions are a very important component of the treatment of children with obesity. In order to maintain the newly learned behavior booster sessions are necessary during the first two years. The child psychologist and the social worker organize the booster sessions. The topics that are repeated are: problem solving techniques and relapse prevention techniques.

## Treatment Control group

The control group is given an initial physical activity and nutritional advice. After 12 months the children are offered the multidisciplinary treatment. During the 12 months study period the children are seen at start, after 3 months and at the end of the period just before they start the treatment.

## Study procedures

### Medical examination

During the first visit to the pediatric clinic general information is collected concerning pregnancy (duration of gestation, smoking, hyperemesis, pre-eclampsy, medication, diabetes) and birth (position of the child during delivery, complications during delivery, birth weight, height, head circumference). Also information on motor and mental development (current educational level) of the child is collected. Furthermore, information on the highest accomplished educational degree and current work of the parents, as well as height, weight and activity level of the parents and other family members and the occurrence of diseases in the family is collected.

At physical examination the occurrence of hirsutism, acanthosis nigricans and possible dysmorphic characteristics is evaluated. Pubertal development is recorded by the pediatrician according to Tanner [87]. Breast development in girls and genital development in boys is used for pubertal classification into two groups. Boys with genital stage 1 and girls with breast stage 1 are classified as prepubertal, and boys and girls with stage 2-5 are classified as pubertal.

### Anthropometric parameters

Weight is measured to the nearest of 0.1 kg using an electronic scale (SECA 911, Vogel & Halke, Hamburg, Germany) and height to the nearest of 0.1 cm with a stadiometer (Holtain, limited, Crymych, Dyfed, Britain) in underwear and barefoot. The BMI is calculated as weight / height squared (kg/m^2^). Subjects are classified as obese using BMI gender- and age specific international cut-off levels developed by Cole et al. [2] BMI expressed as standard deviation score (SDS) for Dutch reference data [82] is used as the main outcome parameter.

Waist circumference (WC, in cm) is measured with an anthropometric tape midway between the lower rib margin and the iliac crest at the end of gentle expiration. The waist to height ratio (WC/Ht) is calculated as WC/height, both measured in centimeters.

Blood pressure measurements (Criton Dinamap, No. 8100) are performed in a relaxed sitting position, in duplicate; the last measurement is used for analyses.

### Blood sample analysis

With the participant in the supine position, blood samples are taken by venipuncture after an overnight fast. Before blood sampling, the fasting state is verbally confirmed by the participant and the parent. Fasting blood samples are taken to determine glucose, insulin, C-peptide, total cholesterol, HDL-cholesterol, LDL-cholesterol, triglycerides, free fatty acids, free T4, TSH and inflammation parameters (CRP, adiponectin).

### Mixed Meal Tolerance Test

The children are asked to consume at least an amount of 150 g of carbohydrates three days prior to the mixed meal tolerance test and continue with their normal daily physical activities. The day before the test the children are asked not to consume any food or drinks after 10 pm, with the exception of tap water.

On the morning of the mixed meal tolerance test the fasting state is verbally confirmed by the participant and the parents. An antecubital intravenous catheter is placed for blood sampling. Fasting blood samples are taken twice with an interval of 15 minutes (t = -15 and t = 0). After the second fasting blood sample is taken, the participant receives a mixed meal bolus of 200 mL (Nutridrink Yoghurt Style, Nutricia, Zoetermeer, The Netherlands). The mixed meal bolus consists of 49% carbohydrates, 35% lipids and 16% proteins. After the consumption of the mixed meal bolus, blood samples are taken two times with 15 minutes intervals (t = 15 and t = 30) and four times with 30 minutes interval (t-60, t = 90, t = 120 and t = 150). Blood samples are analyzed and plasma glucose, plasma insulin and gut hormones (Ghrelin, GLP-1, PYY) are determined. In order to be able to analyze levels of the gut hormones GLP-1 and PYY it is necessary to add the enzyme dipeptidyl peptidase IV (DPPIV) to the tubes.

### Insulin Resistance and Insulin Sensitivity

An index for insulin resistance is calculated according to the Homeostasis Assessment Model for insulin resistance (HOMA-IR) formula: (fasting insulin (mU/mL) × fasting glucose (mmol/L)) / 22.5 [88].

Insulin sensitivity is calculated using the quantitative insulin-sensitivity check index (QUICKI) formula: 1/(log fasting insulin (mU/L)+log fasting glucose (mg/dl)) [89].

### Voluntary Maximal Exercise Test

The physical fitness of the children is determined by a voluntary maximum exercise test on a treadmill using breath-by-breath analysis and plots according to Wasserman [90].

The exercise test consists of three stages; a reference stage (2 minutes rest measurement prior to the exercise test), the test stage (the exercise test till voluntary exhaustion) and a recovery stage (2 minutes recovery measurement in rest after the exercise test). The test stage starts with a velocity of 4 km/h and an angle of 0% during 1 minute. Every minute the velocity is increased with 0.5 km/h and the angle with 2%, till voluntary exhaustion. During the test the participants are encouraged to exert a maximum effort. At the end of the test the children are asked to grade the level of exhaustion by using the Borg scale [91] and the reason to stop.

In adults a maximum effort is achieved when two of the following criteria are fulfilled:

• The difference in heart rate between the last two stages was less than 5 beats per minute.

• The difference in VO_2 _was less than 100 mL/min between the last two stages.

• A Respiratory Exchange Ratio (RER) of > 1.0.

However, the first two criteria are not always found in adults and rarely in children. Therefore, in this study peak values are referred to instead of maximum values. Peak values are reached when the children look physically exhausted and a RER of > 1.0 is accomplished. The physical fitness is calculated from the absolute peak value of oxygen uptake, standardized to age and gender (VO_2peak_-SDS). Yet the absolute peak oxygen uptake in obese can give an overestimation of the real physical fitness because of their increased body size [90]. For that reason also the peak oxygen uptake adjusted for body weight, age and gender (VO_2peak_-SDS-kg) is determined to establish a more realistic value for the physical fitness.

### Health Related Quality of Life

The health related Quality of Life (HRQOL) of the children is determined by the questionnaires DISABKIDS and KIDSCREEN [92]. The DISABKIDS and KIDSCREEN projects developed an instrument with a generic part (KIDSCREEN), and a chronic generic part (DISABKIDS). The KIDSCREEN part of the questionnaire is suitable for children aged between 8-18 years, and the DISABKIDS part for children aged between 4-18 years. The generic part of the questionnaire is used to compare children with a disease with healthy children. For this part only the child version is used. With respect to DISABKIDS, also the parental version is used, to compare the HRQOL reported by the child with the child's HRQOL from the parents' view.

The KIDSCREEN contains 52 questions divided over 10 subscales of 3-7 items each. The DISABKIDS part of the questionnaire contains 37 questions divided over 6 subscales of 6-7 items each. Every question can be answered by choosing from 5 options: never, almost never/ seldom, average/ sometimes, quite often, always. The items are scored on a 5-point scale (0-4). Both parts are scored by summing the responses for the 52 and 37 items, respectively, after adjustment for the negative items, and expressed as percentages between 0-100. A higher score reflects a better HRQOL.

KIDSCREEN is used to compare the HRQOL of the children with obesity with a normal weight control group and DISABKIDS to compare the obese children in the intervention group with the children in the obese control group. The KIDSCREEN questionnaire has demonstrated a Cronbach a reliability coefficient ranging between .77 and .89 for all ten domains [92]. The Cronbach a reliability coefficient of the child version of the DISABKIDS ranges between .70 to.87 for children aged 8-12 years and between .77 to.90 for children aged 13-16 years [92]. A Cronbach a ≥0.7 is considered as good validity.

### Other questionnaires used during the screening phase of the treatment

• *Dutch Eating Behavior Questionnaire (Nederlandse Vragenlijst Eetgedrag/ NVE):*

The NVE [84] is administered during the screening phase. It consists of three subscales: Emotional Eating (13 items), External Eating (10 items), and Restrained Eating (10 items). The items are scored on a 3-point Likert-type scale ranging from *never *to *very often*. The items in the conditional format also have the response option *not relevant*. A high score reflects a specific eating behavior of the particular subscale.

The NVE scales have been proven to provide good internal consistency, satisfactory factorial validity and dimensional stability [72,84].

To assess the child's psychological and social adjustment three versions of the Child Behavior Checklist are asked to fill in. One version is filled in by the parents (Child Behavior Checklist), one by the child themselves (Youth Self Report) and one by a school teacher (Teacher Report Form):

• *Child Behavior Checklist (CBCL):*

The Dutch version of the CBCL [85] is completed during the screening phase by the parents. The questionnaire includes 138 items and yields scores for total behavior problems, internalizing and externalizing behavior of their child. Also three scores for competence are determined (activity, social competence, school competence)

• *Youth Self Report (YSR)*:

The children fill in the YSR [85] version of the CBCL during the screening phase to visualize the child's own view on his or her internalizing and externalizing behavior.

• *Teacher Report Form (TRF)*:

A school teacher of the child fills in the TRF [85] version of the CBCL in order to determine the internalizing and externalizing behavior of the child at school.

• *Perceived Competence Scale for Children (Competentie Belevings Schaal voor Kinderen/ CBSK)*:

The CBSK [86] is used during the screening phase to determine the self-perception or self-concept of the child. The CBSC consists of 28 items and assesses the child's self-perception in four different areas of perceived competence; cognitive ability, physical activity, peer relations, and general self-esteem.

## Type of Analysis

The analyses are performed by using the Statistical Package for Social Science SPSS, v. 17.0 for WINDOWS; SPSS Inc., Chicago, IL, USA) and the level of significance is set to < 0.05, according to an intention-to-treat analysis.

Data are checked for normality before analysis using descriptive statistics and histograms. Data are expressed as mean ± standard deviation (continuous variables) and as count and percentage (categorical variables), unless specified otherwise.

For the analysis of treatment effect between both obese study groups for continuous data, independent t-tests, (M)AN(C)OVA and mixed model for repeated measures are used per time point, adjusted for baseline values. Categorical data are analyzed with the Chi-square test for comparison between groups. In addition, paired t-tests, (M)AN(C)OVA and mixed model for repeated measures are used to compare the parameters within treatment groups over time. For predictive analysis linear and logistic regression analysis is used for continuous dependent variables and binomial variables, respectively.

## Power Calculation

The sample size of a study was based on the power calculation for unpaired means with normal distribution and can be calculated by the following formula:

In this study α = 0.05 and β = 0.20 is chosen, so z_1-α/2 _= 1.96 en z_1-β _= 0.84.

The results of an unpublished pilot study of the proposed treatment given to children with obesity in our hospital showed a clinically relevant difference of 0.6 BMI-SDS between intervention and control group, and the difference between post-treatment and baseline ranged between -2.85 and + 0.70. The difference between the means is therefore chosen as μ_1 _- μ_2 _= 0.6 and the estimated sigma, under normality assumption, will be 3.55/4 = 0.89, so σ_1_^2^ + σ_2_^2^ = 1.58.

In the formula this determines a N_1 _= N_2 _= 35. Considering the likely phenomenon of drop-out a N_1 _= N_2 _= 40 is chosen. The sample size is not adjusted for stratification.

## Burden and risks

For evaluation of short-term, midterm and long-term effects of the treatment, measurements are taken at the beginning of the treatment, after 3 months treatment and after 12 (intervention- and control group) and 24 (intervention group only) months of follow-up. During these three (four) visits a physical examination is performed, cardiovascular fitness measured, a mixed meal test performed and blood samples are taken. Children are also asked to complete a quality of life questionnaire.

## Discussion

The increasing prevalence of childhood obesity and its impact on individuals and society requires effective preventive programs and therapeutic strategies. The study 'Haagse Maatjes': an effect evaluation of a family-based cognitive behavioral multidisciplinary lifestyle intervention, is a randomized controlled trial with a longitudinal design. The study aims to establish long-term weight reduction and stabilization, reduction of obesity related health consequences and improvement of the self-image by a change in lifestyle. In order to adjust to a healthy lifestyle, thinking patterns, level of physical activity and eating behavior of the child with obesity and their family must change. For long term-effect of the treatment it is also important that coping strategies and social skills are taught to children with obesity to deal with low self esteem, low self-control and to teach social skills. Also parental involvement and support are necessary for long-lasting, successful treatment results.

We hypothesize that after the intensive treatment period the intervention group will show a significantly lower BMI-SDS compared to baseline and the control group, and that in subjects with a BMI-SDS decrease of more than 0.5, which is considered a clinically relevant weight reduction [79,81,93], a significant health improvement (secondary study outcomes) is attained.

The design of the study is prospective with follow-up measurements at 3, 12 and 24 months. The longitudinal design of the study makes it possible to perform a predictive analysis, rather than only cross-sectional correlations. Furthermore, as mentioned by the authors of the Cochrane review [77], there is an urgent need for interventions with a psychosocial focus for behavioral change and good clinician-family interaction. We believe that with our study design we do focus on these important aspects.

The children in the intervention group are followed for 2 years and those in the control group for 1 year. Children in the control group are offered the treatment after 1 year, since it is preferable to start treatment of obesity as soon as possible. We expect that some children will drop out of the study during or after the treatment phase. In our power calculation a 10% loss to follow-up was accounted for.

The strength of our study is that, next to the evaluation of the changes in BMI-SDS after intensive lifestyle treatment in obese children, we also assess the effect of weight loss on metabolic health status, inflammation, physical fitness, health-related quality of life and secretion of some gastrointestinal hormones.

The results of this study will provide more insight in the long-term effects of a family-based cognitive behavioral multidisciplinary intervention on weight and BMI-SDS, as well as intended health improvement in children with obesity and their family.

## Abbreviations

BMI: Body Mass Index; BMI-SDS: Body Mass Index Standard Deviation Score; CBCL: Child Behavior Checklist; CBSK: Perceived Competence Scale for Children (CompetentieBelevingsSchaal voor Kinderen); CRP: C-Reactive Protein; GLP-1: Glucagon-like peptide 1; HDL-cholesterol: High Density Lipoprotein Cholesterol; HOMA-IR: Homeostasis Assessment Model for Insulin Resistance; HRQOL: Health related Quality of Life; MS: Metabolic Syndrome; NVE: Dutch Eating Behavior Questionnaire (Nederlandse Vragenlijst Eetgedrag); PYY: Peptide YY; QUICKI: quantitative insulin-sensitivity check index; RER: Respiratory Exchange Ratio; TG: Triglyceride; TRF: Teacher Report Form; VO_2peak_-SDS = Peak oxygen uptake; adjusted for gender and age; VO_2peak_SDS-kg = Peak oxygen uptake; adjusted for gender age and body weight; WC: Waist Circumference; WC/Ht: Waist to Height Ratio; YSR: Youth Self Report

## Competing interests

The authors declare that they have no competing interests.

## Authors' contributions

All authors are responsible for the design of the study and contributed to the intellectual content of the protocol. RCV was responsible for the implementation of the intervention, data collection, data analysis and drafted the study protocol with suggestions and contribution of all other authors. ECAMH obtained financial support. All authors read and approved the final manuscript.
